# Device‐Specific Responses to Pacemaker‐Mediated Arrhythmia in Patients With Prolonged Ventriculoatrial Conduction: A Comparative Simulation Study

**DOI:** 10.1002/joa3.70344

**Published:** 2026-04-23

**Authors:** Mitsuru Tsunomori, Teruhiko Imamura, Daisuke Nagamine, Satoshi Okino, Hitomi Iio, Kuniaki Sato, Naoya Kataoka, Keisuke Uchida, Koichiro Kinugawa

**Affiliations:** ^1^ Medical Equipment Management Center Toyama University Hospital Toyama Japan; ^2^ Second Department of Medicine University of Toyama Toyama Japan

**Keywords:** endless loop tachycardia, pacemaker‐mediated tachycardia, repetitive nonreentrant ventriculoatrial synchrony, ventriculoatrial conduction

## Abstract

**Background:**

Pacemaker‐mediated tachycardia (PMT) triggered by prolonged ventriculoatrial conduction (VAC) can be challenging to detect and terminate. The behavior of device‐specific anti‐PMT algorithms in such scenarios, as well as the potential utility of DDI mode as an alternative strategy, remain to be fully elucidated.

**Methods:**

Five dual‐chamber pacemakers from Abbott, BIOTRONIK, Boston Scientific, Medtronic, and MicroPort CRM were tested using an electrophysiological simulator under VAC times of 450 ms and 550 ms. We assessed (1) PMT detection and termination in DDD mode, (2) atrial sensing within the post‐ventricular atrial refractory period (PVARP) in DDI mode, and (3) the timing adjustment of atrial pacing after retrograde atrial sensing.

**Results:**

At VAC 450 ms, all devices except Medtronic detected PMT. Only Abbott terminated PMT at 550 ms with a shortened AV delay. Four devices detected atrial events within PVARP at 450 ms, whereas only BIOTRONIK did so at 550 ms. Among these, BIOTRONIK, Boston Scientific, and Medtronic adapted atrial pacing timing following atrial sensing within the refractory period, while Abbott did not, resulting in repetitive nonreentrant ventriculoatrial synchrony.

**Conclusions:**

Anti‐PMT performance varies markedly among manufacturers. Devices featuring a longer PVARP and an atrial pacing delay algorithm provide superior protection against pacemaker‐mediated arrhythmias during prolonged VAC. These findings underscore the importance of individualized pacemaker selection and careful programming.

## Graphical abstract

Simulation‐based comparison of five pacemakers reveals that algorithmic differences via manufacturers significantly affect detection and termination of pacemaker‐mediated arrhythmias during prolonged ventriculoatrial conduction. Devices with a longer post‐ventricular atrial refractory period and an atrial pacing delay algorithm offer better protection against these arrhythmias.

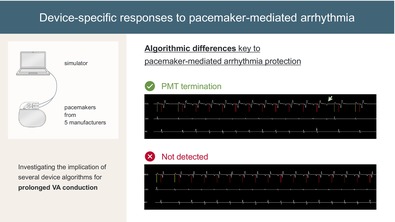

## 1 Background

Cardiac implantable electronic devices (CIEDs), particularly dual‐chamber systems, have become an essential therapeutic modality for patients with atrioventricular (AV) block. However, in individuals with preserved ventriculoatrial conduction (VAC), device‐mediated arrhythmias, most notably pacemaker‐mediated tachycardia (PMT), remain a clinically significant complication [1, 2]. PMT is a reentrant tachycardia perpetuated when the device senses retrograde atrial activity induced by ventricular pacing (VP) and erroneously interprets it as intrinsic atrial activation, thereby initiating a pathological pacing loop. The heart rate during PMT depends on the ventriculoatrial conduction time (VAC time), the programmed maximum tracking rate (MTR), and the sensed AV delay (SAV). Typically, PMT occurs at the MTR of the device when the sum of the VAC time and SAV is less than the pacing interval at the MTR. Conversely, if the sum of the VAC time and SAV is longer than this interval, PMT occurs in cycles below the MTR [3, 4, 5].

Previous studies have demonstrated that VAC is not an uncommon finding in patients undergoing dual‐chamber pacing. Approximately 80% of patients with sick sinus syndrome and 35% of patients with AV block exhibit VAC [6]. Furthermore, 47% of all patients who require pacemaker implants have VA 1:1 conduction; specifically, 67% of those with sinus node dysfunction and 14% of those with complete antegrade block have VA conduction at a mean interval of 235 ± 50 ms (range 110–380 ms) [7]. In a cohort of 250 patients who received CIEDs for AV block, VAC was observed in approximately 30% of cases (*n* = 76), with a wide range of VAC time (180–440 ms; mean 258 ± 65 ms) [8]. However, the prevalence of patients with prolonged VAC (e.g., exceeding 400 ms) has not been clearly established. Notably, prolonged VAC can significantly impair the ability of CIEDs to detect and appropriately terminate PMT events [8].

To prevent PMT, setting a post‐ventricular atrial refractory period (PVARP) longer than VAC time is effective. However, if the VAC falls within the PVARP and the atrium is paced while still in the atrial refractory period, it must not be captured. A sequence characterized by functional atrial undersensing and subsequent non‐capture of atrial pacing is known as repetitive nonreentrant ventriculoatrial synchrony (RNRVAS). Factors that cause RNRVAS include high AV sequential pacing rates, long programmed AV delay, and long PVARP [4, 9].

Modern CIEDs are equipped with manufacturer‐specific algorithms designed to identify and interrupt PMT episodes. These algorithms generally rely on the detection of consistent atrial sensing (AS) occurring at regular intervals following VP to initiate corrective measures, such as temporary mode switching or PVARP extension. However, when retrograde conduction is markedly delayed, such that the VAC time exceeds the detection threshold of anti‐PMT algorithms, retrograde P waves may be erroneously interpreted as sinus activity, failing to detect PMT or to initiate appropriate corrective measures [3, 10]. If PMT cannot be avoided by adjusting the PVARP or utilizing anti‐PMT algorithms, an alternative strategy is to change the pacing mode from DDD to DDI [3, 5, 11]. Nevertheless, the appropriateness of switching to DDI in the presence of prolonged VAC, particularly regarding risks such as inducing RNRVAS, has not been systematically evaluated.

Furthermore, atypical forms of PMT, characterized by a heart rate lower than MTR associated with long or ultra‐long VA conduction, have been increasingly recognized. These episodes are often refractory to conventional device programming strategies, because they may not satisfy the detection criteria of standard anti‐PMT algorithms. In such scenarios, electrophysiological interventions including targeted ablation of the retrograde AV nodal pathway may be required [12, 13]. However, optimal strategies to manage individuals with long or ultra‐long VA conduction remain unestablished.

Therefore, two key issues remain unresolved. First, the behavior of manufacturer‐specific anti‐PMT algorithms when confronted with prolonged VAC needs to be clarified; and second, whether switching from DDD to DDI is a reasonable therapeutic option when anti‐PMT algorithms fail warrants evaluation. Given the difficulty of identifying and reproducing the response of individual algorithms to pacemaker‐mediated arrhythmias arising from significantly prolonged VAC in a clinical setting, we utilized an established simulator in the present study. Specifically, we investigated the response and limitations of several device algorithms under conditions of prolonged VAC and assessed the potential utility of DDI mode as an alternative programming strategy.

## 2 Methods

### 2.1 Simulation Setup and Clinical Scenario Reproduction

A programmable electrophysiological simulator (RSIM‐1500‐USB Device‐Interactive Simulator System, Rivertek Medical Systems, Ireland) was employed to reproduce clinical arrhythmia conditions. The scenarios included sinus arrest, complete AV block, and VAC with intervals of 450 and 550 ms. These VAC times were selected based on the maximum value (440 ms) observed in a prior cohort study and a conduction time (520 ms) reported in a case report [8, 11]. Notably, the actual VAC time measured by each pacemaker is influenced by factors such as the device's sensing filter characteristics and may differ slightly from the value set in the simulator. The simulator provided programmable intracardiac signals and enabled real‐time pacing and sensing interactions between the test devices and simulated rhythms.

### 2.2 Devices Under Investigation

Five dual‐chamber pacemakers from different manufacturers were investigated under identical conditions: (1) Assurity MRI/2272 (Abbott Laboratories, Abbott Park, Illinois, USA); (2) Edora 8 DR‐T/407 145 (BIOTRONIK SE & Co. KG, Berlin, Germany); (3) ACCOLADE MRI EL/L331 (Boston Scientific Corporation, Marlborough, Massachusetts, USA); (4) Azure XT DR MRI/W2DR01 (Medtronic plc, Minneapolis, Minnesota, USA); (5) ALIZEA DR 1600/TPM020C (MicroPort CRM, Clamart, France). Each device was evaluated for specific functional responses based on the following protocols.

### 2.3 Protocols and Evaluation Parameters

#### 2.3.1 (1) Detection and Termination of PMT

To evaluate the limits of anti‐PMT algorithms, this study assessed whether device‐specific algorithms functioned correctly even under prolonged VAC time. Each device was programmed in DDD mode with a lower rate limit of 70 ppm, MTR of 130 ppm, SAV of 140 ms, and paced AV delay (PAV) of 200 ms (205 ms for MicroPort CRM). PMT was induced by simulating retrograde P waves. The ability of each device to detect and appropriately terminate PMT was assessed under both VAC of 450 ms and 550 ms.

#### 2.3.2 (2) Atrial Sensing Within the PVARP

This protocol aimed to determine whether retrograde P waves could be recognized as atrial sensing within the refractory period (AR) when utilizing DDI mode—a potential alternative strategy when anti‐PMT algorithms are ineffective. Each device was reprogrammed to DDI mode at 70 ppm. Except for the MicroPort CRM device, PVARP was extended to its maximum programmable value. The presence or absence of AR was documented for each device.

#### 2.3.3 (3) Adjustment of Atrial Pacing After AR

Since atrial pacing (AP) occurring in proximity to retrograde P waves may not capture the atrium due to the atrial refractory period, this protocol investigated whether the devices incorporate logic to adjust the timing of the subsequent AP. In cases where AR occurred, the timing of the subsequent AP was analyzed to assess whether the device appropriately delayed the pacing stimulus to avoid competition with retrograde atrial contraction caused by VAC. Protocols (2) and (3) evaluated features designed to minimize the risk of arrhythmogenic interaction via RNRVAS.

### 2.4 Statistics

Due to the qualitative nature of the device algorithm behaviors in response to simulated events, most comparisons were descriptive. For each device under both VAC settings, the following were recorded as categorical outcomes: the presence or absence of PMT detection and termination, any required parameter changes to achieve successful detection, the recognition of AR, the timing adjustment of AP following AR, and the occurrence of RNRVAS.

## 3 Results

The differences in responses to PMT and VAC are summarized in Table 1 and reported below, supported by electrograms recorded from the simulator and device programmers.

**TABLE 1:** Summary of device‐specific responses to simulated pacemaker‐mediated tachycardia and retrograde atrial conduction.

	Abbott (assurity MRI)	Biotronik (edora 8 DR‐T)	Boston scientific (accolade MRI EL)	Medtronic (azure XT DR MRI)	MicroPort CRM (alizea DR 1600)
Study 1					
VAC 450 ms					
PMT detection	Yes	Yes	No	No	Yes
PMT termination	Yes	Yes	No	No	Yes
Required changes	PMT Detection Rate ≤ 95 bpm	VA criterion to 500 ms	MTR ≤ 95 bpm		
VAC 550 ms					
PMT detection	No	No	No	No	No
PMT termination	No	No	No	No	No
Required changes	PMT Detection Rate to 90 bpm and SAV shortening ≤ 90 ms		MTR ≤ 80 bpm (non‐terminated: VAC exceeded PVARP)		
Study 2					
VAC 450 ms					
AR detection	Yes	Yes	Yes	Yes	No
VAC 550 ms					
AR detection	No	Yes	No	No	No
Study 3					
AP delay after AR	No	Yes (shortened PAV)	Yes (shortened PAV)	Yes (alternating PAV)	N/A
RNRVAS occurrence	Yes	No	No	No	N/A

*Note:* This table summarizes the behavior of five dual‐chamber pacemakers from different manufacturers under simulated conditions with ventriculoatrial conduction (VAC) time of 450 ms and 550 ms. Devices were assessed for: (1) the detection and termination of pacemaker‐mediated tachycardia (PMT) in DDD mode; (2) the ability to sense atrial events within the refractory period (AR) during the post‐ventricular atrial refractory period (PVARP) in DDI mode; and (3) the adjustment of atrial pacing (AP) timing following AR detection. PMT detection depended upon algorithm‐specific criteria such as PMT Detection Rate, VA criterion, and Maximum Tracking Rate (MTR). AR detection and AP delay behavior varied across manufacturers, with some devices demonstrating dynamic AV delay shortening or alternating pacing intervals.

Abbreviations: AP, atrial pacing; AR, atrial sensing within the refractory period; LRL, lower rate limit; N/A, not applicable; PAV, paced AV delay; PMT, pacemaker‐mediated tachycardia; RNRVAS, repetitive nonreentrant ventriculoatrial synchrony; SAV, sensed AV delay; VAC, ventriculoatrial conduction.

### 3.1 Detection and Termination of PMT in Each Device (Study 1)

The ability to detect and terminate PMT under VAC 450 ms varied by device, with results as follows: algorithm names in parentheses.

#### 3.1.1 Abbott ( **PMT** Response)

**FIGURE 1:**
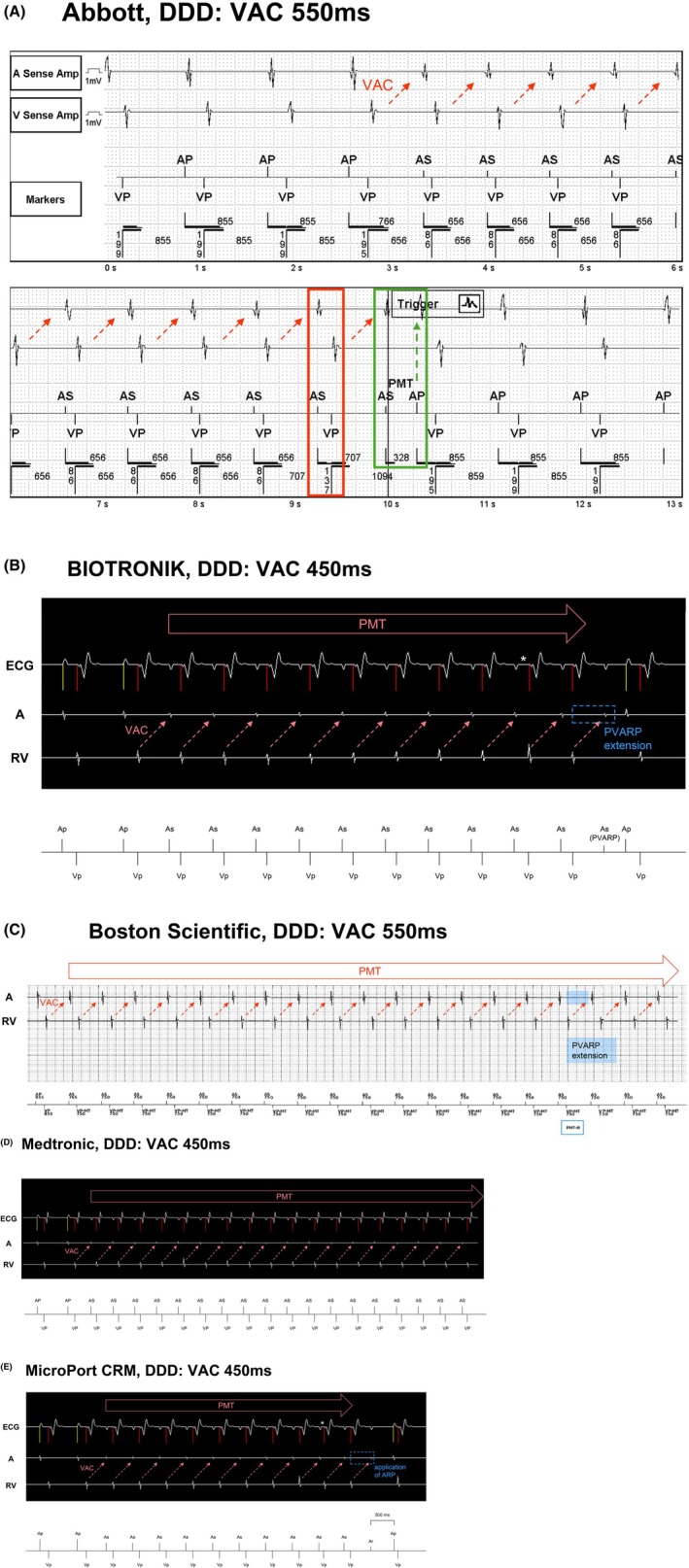
(A) PMT Response (Abbott). This figure shows a pacemaker‐mediated tachycardia (PMT) episode stored in the memory of the Abbott device. The ventriculoatrial conduction (VAC) that occurred after the fourth ventricular pacing (VP) triggered the PMT. After 8 beats with A‐A intervals faster than the set *PMT detection rate*, the device evaluates the stability of the interval between VP and subsequent atrial sensing (AS). If this interval is stable and remains constant even when the next sensed AV delay is modulated (red rectangle), the device detects the presence of PMT. The device suppresses VP and terminates PMT with atrial pacing (AP) after AS (green rectangle). To detect PMT induced under VAC 550 ms, the sensed AV delay should be set short, and the actual PMT rate must be above the set *PMT detection rate*. (B) PMT Protection (BIOTRONIK). If the VP‐AS interval is shorter than the *VA criterion* for 8 consecutive beats and the deviation is small, the device checks whether changing the sensed AV delay (asterisk) on the next beat affects the VAC time. If it does not change, the next post‐ventricular atrial refractory period (PVARP) is extended 50 ms beyond the *VA criterion*, terminating the PMT when the retrograde P wave falls within the PVARP (blue rectangle). Ap: Atrial pacing, As: Atrial sensing, As (PVARP): Atrial sensing within the PVARP, Vp: Ventricular pacing. (C) PMT Termination (Boston Scientific). To detect PMT, 16 consecutive VP cycles at the maximum tracking rate (MTR) after AS (VP‐MT in the figure) are counted, and the 16‐beat VAC time must fall within a 32 ms range based on the VAC time starting from the second VP‐MT. Unlike other devices, the detection algorithm does not require the VAC time to remain unchanged when the sensed AV delay is varied during PMT detection. The above conditions are met, and the PVARP is extended to 500 ms (blue shading) to terminate the PMT at PMT‐B in the figure. However, VAC 550 ms extends beyond this window, and the PMT continues. AP: Atrial pacing, AS: Atrial sensing, VP: Ventricular pacing, VP‐MT: Ventricular pacing at the maximum tracking rate, PMT‐B: PMT brake operation. (D) PMT not detected (Medtronic). A VAC extending beyond 400 ms cannot be detected by Medtronic devices; consequently, the PMT is sustained. AP: Atrial pacing, AS: Atrial sensing, VP: Ventricular pacing. (E) Anti‐PMT (MicroPort CRM). The interval between 8 cycles of VP and AS must be stable within 470 ms. Secondly, the VAC time is checked for stability even after modulating the AV delay (asterisk). If it is determined to be PMT, an atrial refractory period of 500 ms (so‐called PVARP) is applied after the next VP (blue rectangle). This results in the VAC being sensed within the device's refractory period, and the PMT is terminated. The observed 500 ms interval from the retrograde P wave to AP appears similar to the 500 ms atrial escape interval triggered by sensing within WARAD, a feature of the MicroPort CRM device. Ap: Atrial pacing, As: Atrial sensing, Ar: Atrial sensing during a relative refractory period, Vp: Ventricular pacing, ARP: Atrial refractory period.

This device includes a programmable *PMT Detection Rate*. The PMT induced under VAC 450 ms (measured at 468 ms by the pacemaker) had a tachycardia cycle length of approximately 605 ms (slightly under 100 bpm). When the PMT Detection Rate was set below 95 bpm, the device successfully detected and terminated the PMT. The minimum programmable Detection Rate is 90 bpm. Under VAC 550 ms (measured at 570 ms by the pacemaker) and SAV 140 ms, the PMT cycle length extended to 710 ms (84.5 bpm) and thus fell below the detection threshold; however, when the SAV was shortened to 90 ms, the cycle length became faster than the detection rate, allowing for successful detection and termination (Figure 1A).

#### 3.1.2 **BIOTRONIK** ( **PMT** Protection)

The algorithm uses the *VA criterion* as the detection threshold. The maximum programmable VA criterion is 500 ms. Therefore, PMT with VAC 450 ms was successfully detected and terminated (Figure 1B). However, PMT with VAC 550 ms could not be detected due to the VAC time exceeding the detection limit.

#### 3.1.3 Boston Scientific ( **PMT** Termination)

Detection required the PMT to persist at the *Maximum Tracking Rate (MTR)*. The device could detect PMT if the MTR was set at 95 bpm for VAC 450 ms, and 80 bpm for VAC 550 ms. This device terminates PMT by extending the PVARP to 500 ms; however, with VAC 550 ms, retrograde atrial signals fell outside the PVARP, resulting in a failure to terminate the PMT (Figure 1C).

#### 3.1.4 Medtronic ( **PMT** Intervention)

This device failed to detect PMT under both VAC conditions, as its detection algorithm is limited to the VAC time shorter than 400 ms (Figure 1D) [14].

#### 3.1.5 **MicroPort CRM** (Anti‐ **PMT** )

PMT was detected and terminated only under VAC 450 ms (Figure 1E). The algorithm is limited to detecting VAC times shorter than or equal to 470 ms; thus, it failed to detect the PMT at VAC 550 ms [15].

### 3.2 Atrial Sensing Within the Refractory Period (AR Detection) (Study 2)

**FIGURE 2:**
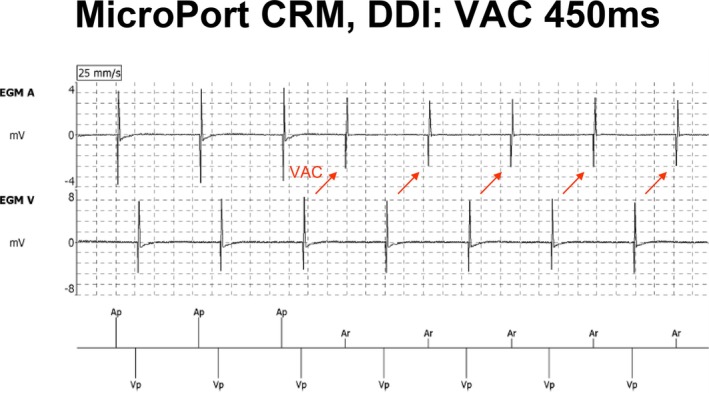
Operation similar to VVI. Although the VAC is marked as “Ar”, the MicroPort CRM's WARAD feature does not function in DDI mode; therefore, it is effectively atrial sensing. As a result, atrial pacing is inhibited, and the AV interval is determined by the VAC time and the lower rate limit, resulting in VVI‐like operation. Ap: Atrial pacing, Ar: Atrial sensing in DDI mode, Vp: Ventricular pacing.

Four devices—Abbott, BIOTRONIK, Boston Scientific, and Medtronic—successfully detected retrograde atrial events within the extended PVARP under VAC 450 ms, confirming appropriate atrial sensitivity to retrograde conduction. MicroPort CRM displays “Ar” for both VAC of 450 ms and 550 ms; however, unlike the AR detection algorithms of other manufacturers, these “Ar” events function effectively as atrial sensing in terms of pacing inhibition (Figure 2). Although MicroPort CRM utilizes the Window of Atrial Rate Acceleration Detection (WARAD) instead of PVARP to monitor early atrial excitation [16, 17], the inactivity of WARAD in DDI mode causes all atrial sensing events to be marked as “Ar” [18, 19]. If these events were strictly processed within the refractory period, the device would deliver atrial pacing at the programmed lower rate limit. As shown in Figure 2, the absence of “Ap” following “Ar” confirms that the device logic treats these “Ar” markers as functional AS events rather than refractory signals.

Only the BIOTRONIK device was able to detect AR under the longer VAC condition of 550 ms, demonstrating superior sensitivity to delayed retrograde atrial activation.

### 3.3 Adjustment of Atrial Pacing Following AR Detection (Study 3)

Among the four devices that detected AR at VAC 450 ms, three devices—BIOTRONIK, Boston Scientific, and Medtronic—delayed the subsequent AP in response to AR, thereby avoiding competition with retrograde atrial activity.

**FIGURE 3:**
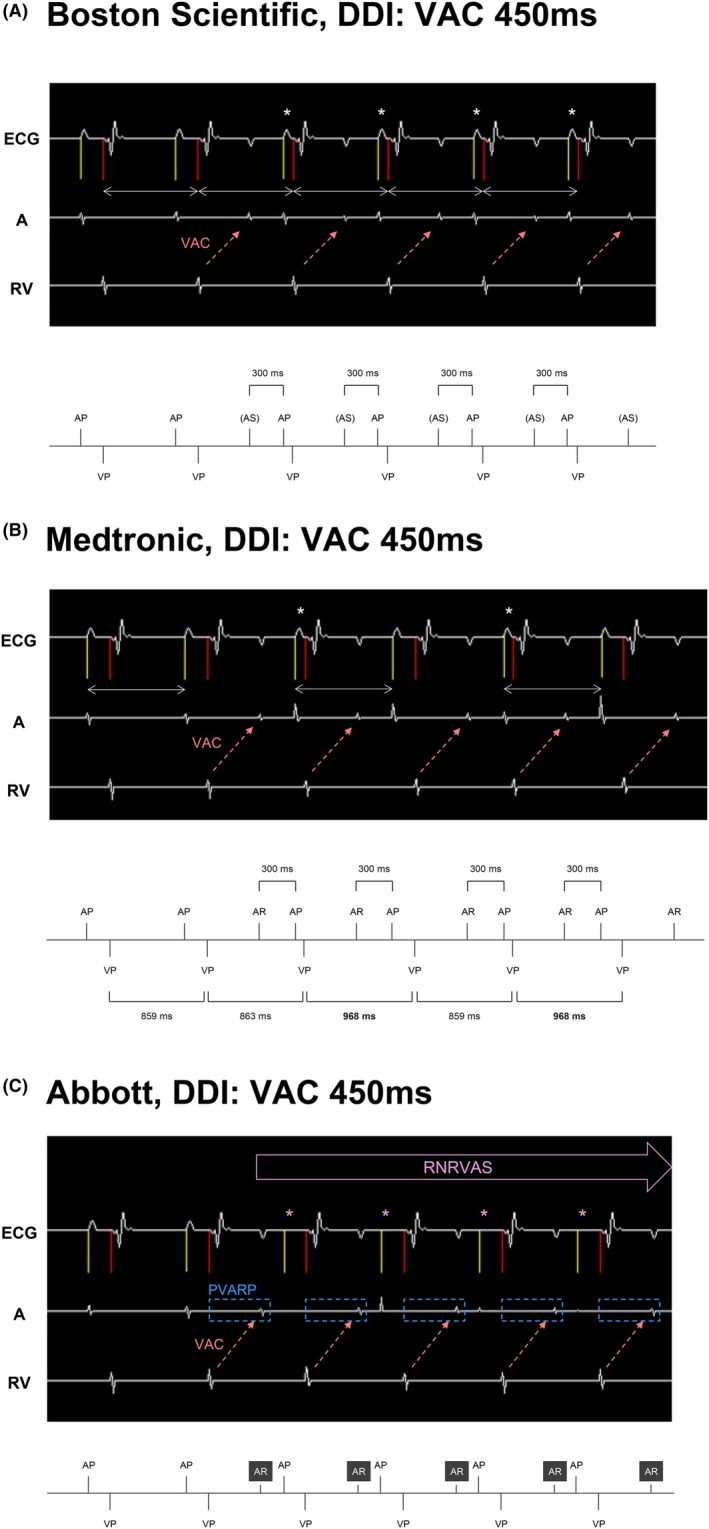
(A) Uniformly shortened PAV after the delayed AP. The APs following the VAC are spaced at regular intervals. The paced AV delay (PAV) after the delayed AP (asterisks) is shorter than the programmed one. In devices using V‐V timing, the V‐V intervals (double‐headed arrows) remain constant. AP: Atrial pacing, AS: Atrial sensing within the PVARP, VP: Ventricular pacing. (B) The PAV varied alternately with every other beat. After the AP delay, the PAV alternates between shortened beats (asterisks) and programmed beats, with intermittent V‐V interval prolongation. The AP‐AP interval of the beat with the prolonged V‐V interval (double‐headed arrows) matched the set lower rate limit, and the following PAV was as programmed, suggesting that it was actuated by A‐A timing. AP: Atrial pacing, AR: Atrial sensing within the PVARP, VP: Ventricular pacing. (C) RNRVAS due to lack of post‐AR AP delay function. When the retrograde P wave and AP are in proximity, the stimulus is ineffective (asterisks) as it falls during the atrial refractory period. Devices without the post‐AR AP delay function will pace at the lower rate limit unless AS is counted. During the PAV and VAC time, the atrium recovers from the refractory period and repeats the VAC. AP: Atrial pacing, AR: Atrial sensing within the PVARP, VP: Ventricular pacing.

In the BIOTRONIK and Boston Scientific devices, VP occurred with a shorter PAV than programmed, following the delayed AP (Figure 3A).

In the Medtronic device, the PAV varied alternately every other beat, showing a dynamic pacing pattern (Figure 3B).

In contrast, the Abbott device lacked a post‐AR AP delay mechanism. Consequently, the immediate AP delivery following AR resulted in RNRVAS—a pseudo‐arrhythmic state caused by atrial pacing into the atrial refractory period (Figure 3C).

## 4 Discussion

In this simulation‐based study, we evaluated the response of five commercially available dual‐chamber pacemakers to prolonged VAC, focusing on their ability to detect and terminate PMT, sense retrograde atrial activation, and adapt AP timing accordingly. Our findings highlight significant differences among manufacturers in the performance of their anti‐PMT algorithms, with important clinical implications.

### 4.1 PMT Detection and Termination

Although PMT termination typically relies on the device's ability to detect retrograde conduction and interrupt the reentrant circuit, our results indicate that this capacity is highly dependent on each manufacturer's algorithmic limitations and programmable parameters. The Abbott device, for example, was able to detect PMT at VAC 450 ms when the PMT Detection Rate was set appropriately. Furthermore, it could detect PMT at VAC 550 ms when the SAV was shortened to 90 ms, suggesting a degree of adaptability in rate and AV timing programming.

In contrast, BIOTRONIK could only detect PMT if the VA criterion was within its maximum setting of 500 ms, thereby missing episodes with longer VAC. Boston Scientific required the PMT to persist at or above the MTR to initiate detection and attempted termination by PVARP extension. However, PVARP extension alone was insufficient for PMT termination when the VAC exceeded 500 ms. Medtronic and MicroPort CRM devices failed to detect PMT at VAC 550 ms, constrained by internal detection thresholds (e.g., VAC time < 400 ms and ≤ 470 ms, respectively). These findings are consistent with prior reports that underscore the challenge of managing PMT in the context of prolonged VAC time [10, 13].

### 4.2 Atrial Sensing Within the Refractory Period and Pacing Adaptation

Atrial sensing within the refractory period (i.e., AR) plays a key role in recognizing retrograde atrial events and coordinating subsequent AP. Four of the five tested devices (excluding MicroPort CRM) successfully detected AR at VAC 450 ms, with only BIOTRONIK maintaining detection at 550 ms, likely due to its extended PVARP capability (up to 600 ms), which exceeds other manufacturers' limits.

Among AR‐positive devices, BIOTRONIK, Boston Scientific, and Medtronic demonstrated an appropriate AP delay following AR. These post‐AR AP delays are essential to avoid atrial non‐capture during refractoriness and to prevent RNRVAS. The Abbott device lacked this adaptive delay, resulting in RNRVAS—a known manifestation of suboptimal atrial timing management [4].

### 4.3 Algorithmic Behavior and Rate Control Implications

Device‐specific post‐AR pacing behaviors also affected AV delay modulation and overall rhythm regularity. Medtronic exhibited alternating PAV intervals, consistent with a design that switches between a short and a standard AV delay in response to sensed atrial activity. This behavior aligns with the Non‐Competitive Atrial Pacing (NCAP) algorithm, which is designed to avoid pacing during atrial vulnerability but may inadvertently create variability in pacing intervals [20, 21, 22].

**FIGURE 4:**
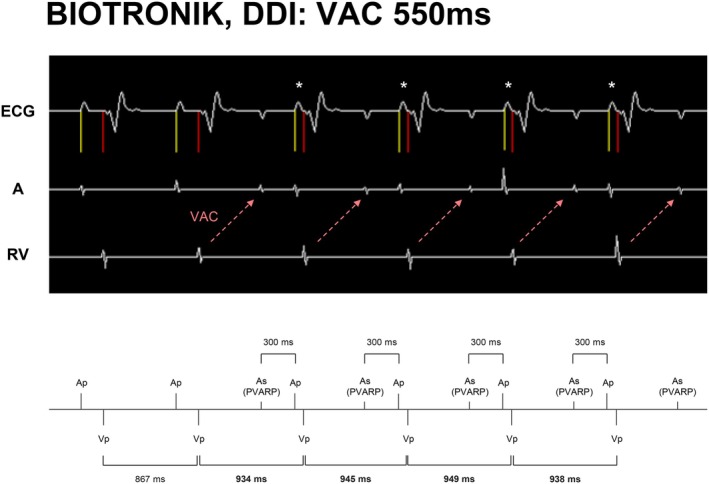
Pacing cycle prolonged beyond the lower rate limit. This figure shows the AP delays following atrial sensing within the refractory period (AR) in the BIOTRONIK device. It is standard behavior to skip the AP and proceed directly to the AR‐VP sequence if the AP delay would prolong the V‐V interval. However, in BIOTRONIK devices, if the sum of the VAC time, post‐AR AP delay, and the shortest PAV (asterisks, 75 ms) exceeds the programmed lower rate limit, the pacing cycle is prolonged beyond the lower rate limit. Ap: Atrial pacing, As (PVARP): Atrial sensing within the PVARP, Vp: Ventricular pacing.

In contrast, Boston Scientific and BIOTRONIK appeared to maintain a more stable V–V interval due to their reliance on V–V timing. However, BIOTRONIK's AP delay mechanism appears to function through A–A timing, which may lead to inappropriate heart rate suppression under conditions of sustained long VAC, especially when the lower rate limit is set high (Figure 4). These interactions highlight the complex dependence of pacing behavior on internal device timing architectures [20, 21, 22].

### 4.4 Clinical Implications and Programming Considerations

Given the diverse behavior of anti‐PMT algorithms, clinicians must be aware of each device's limitations and programming flexibility. If a patient exhibits prolonged VAC exceeding 400 ms, avoiding PMT with the anti‐PMT algorithm may be difficult, depending on the chosen device. BIOTRONIK allows the PVARP to be set up to 600 ms, potentially detecting even significantly prolonged VAC. Furthermore, Abbott can detect and terminate even slow‐rate PMT and record episode waveforms, which may be beneficial for younger patients who are prone to sinus tachycardia and potential PMT. For patients prone to long VAC times, the following strategies are recommended:

1. Set the PVARP to be 50–75 ms longer than the VAC time (excluding MicroPort CRM) to encompass delayed retrograde P waves [8]. In BIOTRONIK, setting the PVARP to 600 ms prevented PMT induction even at VAC 550 ms. However, a long PVARP limits the MTR and carries the risk of sudden 2:1 block when a sinus tachycardia P wave falls within the PVARP. Therefore, the total atrial refractory period should not be unnecessarily prolonged, taking the patient's activity level into account.
2. Unlike long PVARP, anti‐PMT algorithms (except for Boston Scientific) do not affect operation outside of PMT episodes. However, specific parameters must be set appropriately to detect PMT: the PMT Detection Rate for Abbott, the VA criterion for BIOTRONIK, and the MTR for Boston Scientific. Importantly, caution is required when lowering these thresholds to accommodate prolonged VAC times. Overly sensitive settings increase the risk of misidentifying sinus tachycardia or atrial tachycardia as PMT. Boston Scientific's algorithm does not include a verification phase, such as modulating the AV delay to confirm the stability of VAC times, which may result in lower specificity for PMT detection [23]. Additionally, since the Wenckebach phenomenon occurs when the sinus rate exceeds the MTR, setting the MTR too low for PMT detection may unintentionally limit the heart rate response during exercise [16, 23].
3. Consider switching to **DDI mode** in refractory cases, as this eliminates atrial tracking and disrupts the PMT circuit regardless of VAC duration [10]. However, if the VAC is outside of the PVARP, VP will not track the atrial event due to DDI mode; however, the event will be recorded as “AS”. In MicroPort CRM, this is displayed as “Ar” but functions as sensing during non‐refractory period. It should be noted that atrial pacing does not occur until VAC is interrupted (i.e., the VP‐AS sequence continues), effectively resulting in VVI operation.
4. Utilize AP delay functions (e.g., Atrial Upper Rate in BIOTRONIK, Atrial Flutter Response in Boston Scientific, and NCAP in Medtronic) to prevent atrial pacing conflicts. Additionally, atrial extrasystoles may serve as a non‐invasive method to terminate ongoing PMT episodes, especially when timed appropriately during retrograde conduction [24]. However, for devices without an AP delay function, a long PVARP and an increase in the atrial pacing rate (e.g., a high lower rate setting or Rate Response function) can induce RNRVAS. In these cases, the PVARP and the timing of atrial pacing must be adjusted to ensure an adequate atrial refractory period.

If pacemaker‐mediated arrhythmias remain difficult to suppress with the above setting changes, or if the patient's exercise tolerance requires an increase in their maximum heart rate, alternative solutions such as catheter ablation should be considered.

### 4.5 Limitations

This study has several limitations. First, the investigation was conducted using a simulation‐based model. While the RSIM‐1500‐USB simulator offers high‐fidelity replication of intra‐cardiac signals, it may not fully reproduce the complex and dynamic physiological conditions seen in vivo, such as autonomic tone fluctuations, variable AV conduction properties, or fusion/pseudofusion beats. Therefore, the clinical behavior of each device may differ from what was observed in the simulated environment.

Second, the precise incidence of markedly prolonged VAC in a real‐world setting has not been fully established. While our study addresses device behavior in such scenarios, the overall clinical impact and the proportion of patients who might benefit from these specific considerations are difficult to estimate without further epidemiological data.

Third, device‐specific responses were assessed under uniform programming conditions. While this allows standardized comparison, it does not fully reflect individualized clinical programming strategies. For instance, the detection or suppression of PMT might be optimized by adjusting additional parameters such as mode switching criteria, MTR, or atrial sensitivity settings, which were not systematically varied in this study.

Fourth, the study focused solely on dual‐chamber pacemakers from five manufacturers. Results may not be generalizable to ICD, biventricular, single‐chamber, or leadless devices, which may have different algorithmic behaviors and pacing logic. Furthermore, the devices tested represented specific models, and newer firmware or product lines may incorporate updated anti‐PMT features not captured here.

Fifth, no in vivo validation was performed. While prior literature supports the relevance of simulated arrhythmia testing [3, 24], prospective clinical studies are warranted to confirm how the observed behaviors impact real‐world outcomes such as arrhythmia burden, patient symptoms, and battery longevity.

Lastly, this study did not assess the impact of long‐term programming changes, patient‐specific anatomical considerations (e.g., atrial size, lead positioning), or comorbid conditions such as atrial fibrillation, which may significantly affect pacing behavior and therapeutic outcomes.

## 5 Conclusion

This study demonstrated notable differences among pacemaker manufacturers in managing pacemaker‐mediated arrhythmias caused by prolonged VAC. Devices with longer programmable PVARP and post‐atrial sensing delay functions, such as those from BIOTRONIK, Boston Scientific, and Medtronic, were more effective in preventing PMT and avoiding pseudo‐arrhythmias like RNRVAS. In contrast, devices without such adaptations may require careful programming adjustments or non‐tracking modes (e.g., to DDI) to prevent tachycardia.

Understanding each device's algorithmic behavior is essential for optimizing therapy in patients with long VA conduction. Tailored programming based on individual conduction patterns may improve outcomes. Further clinical validation is needed.

## Funding

The authors have nothing to report.

## Conflicts of Interest

The authors declare no conflicts of interest.

## Data Availability

The data that support the findings of this study are available from the corresponding author upon reasonable request.
